# Supplementary material for: Causal Relationship between Aging and Anorexia Nervosa: A White-Matter-Microstructure-Mediated Mendelian Randomization Analysis
Source: Biomedicines. 2024 Aug 16;12(8):1874. doi: 10.3390/biomedicines12081874 (PMC11351342; doi:10.3390/biomedicines12081874)
Supplement: Supplementary file 1 [file biomedicines-12-01874-s001.zip › Supplementary Figure/Figure S4/Singe SNP plot of MR analysis for TL to ubm-b-1708.pdf]

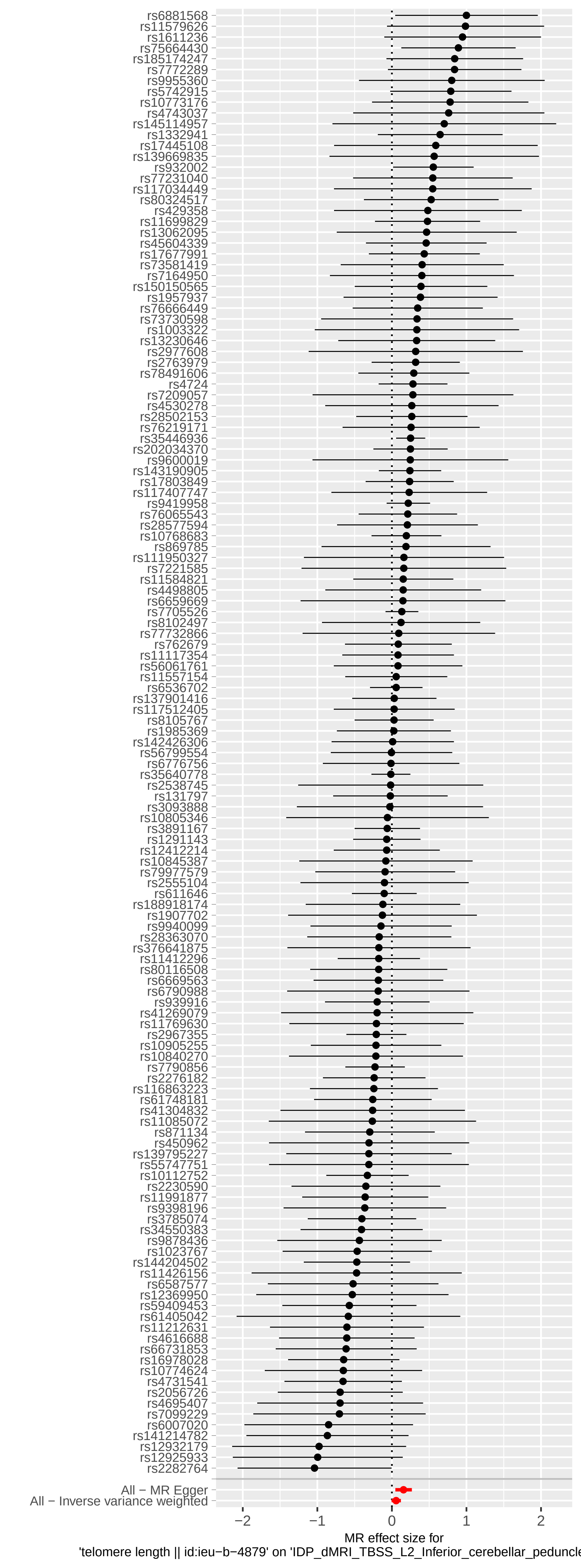
rs6881568
rs11579626
rs1611236
rs75664430
rs185174247
rs7772289
rs9955360
rs5742915
rs10773176
rs4743037
rs145114957
rs1332941
rs17445108
rs139669835
rs932002
rs77231040
rs117034449
rs80324517
rs429358
rs11699829
rs13062095
rs45604339
rs17677991
rs73581419
rs7164950
rs150150565
rs1957937
rs76666449
rs73730598
rs1003322
rs13230646
rs2977608
rs2763979
rs78491606
rs4724
rs7209057
rs4530278
rs28502153
rs76219171
rs35446936
rs202034370
rs9600019
rs143190905
rs17803849
rs117407747
rs9419958
rs76065543
rs28577594
rs10768683
rs869785
rs111950327
rs7221585
rs11584821
rs4498805
rs6659669
rs7705526
rs8102497
rs77732866
rs762679
rs11117354
rs56061761
rs11557154
rs6536702
rs137901416
rs117512405
rs8105767
rs1985369
rs142426306
rs56799554
rs6776756
rs35640778
rs2538745
rs131797
rs3093888
rs10805346
rs3891167
rs1291143
rs12412214
rs10845387
rs79977579
rs2555104
rs611646
rs188918174
rs1907702
rs9940099
rs28363070
rs376641875
rs11412296
rs80116508
rs6669563
rs6790988
rs939916
rs41269079
rs11769630
rs2967355
rs10905255
rs10840270
rs7790856
rs2276182
rs116863223
rs61748181
rs41304832
rs11085072
rs871134
rs450962
rs139795227
rs55747751
rs10112752
rs2230590
rs11991877
rs9398196
rs3785074
rs34550383
rs9878436
rs1023767
rs144204502
rs11426156
rs6587577
rs12369950
rs59409453
rs61405042
rs11212631
rs4616688
rs66731853
rs16978028
rs10774624
rs4731541
rs2056726
rs4695407
rs7099229
rs6007020
rs141214782
rs12932179
rs12925933
rs2282764
All − MR Egger
All − Inverse variance weighted
−2
−1
0
1
2
MR effect size for
'telomere length || id:ieu−b−4879' on 'IDP_dMRI_TBSS_L2_Inferior_cerebellar_peduncle
